# Ideal water temperature environment for giant Marimo (*Aegagropila linnaei*) in Lake Akan, Japan

**DOI:** 10.1038/s41598-023-43792-6

**Published:** 2023-10-06

**Authors:** Keisuke Nakayama, Katsuaki Komai, Motoshi Amano, Shintarou Horii, Yuichiro Somiya, Etsuko Kumamoto, Yoichi Oyama

**Affiliations:** 1 Graduate School of Engineering, Kobe University, Kobe, 657-8501 Japan; 2 Faculty of Engineering, Kitami Institute of Technology, Kitami, 090-8507 Japan; 3 Center of Radiology and Radiation Oncology, Kobe University Hospital, Kobe, Japan; 4 Graduate School of System Informatics, Kobe University, Kobe, 657-8501 Japan; 5 Marimo Research Center, Kushiro Board of Education, Kushiro, 085-0467 Japan

**Keywords:** Ecosystem ecology, Environmental impact, Hydrology

## Abstract

*Aegagropila linnaei* is a filamentous green algal species that often forms beautiful spherical shapes called "lake balls" or "Marimo". *A. linnaei* were once globally distributed around the world, but the population has been declining for several decades. Lake Akan, in Japan, is now the only lake in the world with a colony of giant Marimo (over 20 cm in diameter). Here we show the net growth rate of Marino resulting from photosynthesis and decomposition based on laboratory experiments, MRI analysis, and quantitative element analysis, which show the decomposition rate, the maximum annual Marimo diametric growth rate, and the carbon-to-nitrogen ratio, respectively. We found an explicit dependence of the decomposition rate of Marimo on the cumulative water temperature, with a threshold of 7 °C. MRI analysis showed a high correlation among a Marimo's diameter, surface thickness, and annual diametric growth rate. Moreover, the C/N ratio was high in the exterior side of the surface thickness, indicating that this layer is the main growth area for photosynthesis. These results suggest that the central cavity and the surface thickness represent the change in the growth environment such as water temperature and light intensity. Between the 1980s and the present, Between the 1980s and the present, the cumulative water temperature has increased from about 1250 to about 1600 °C-days. Therefore, the maximum surface thickness has decreased by approximately 1 cm, as estimated by water temperature records and annual diametric growth rates^10^. As a measure to preserve preferable conditions for colonies of giant Marimo in the face of global warming, the flow of low-temperature river water into Marimo colonies should be protected.

## Introduction

**Figure 1:**
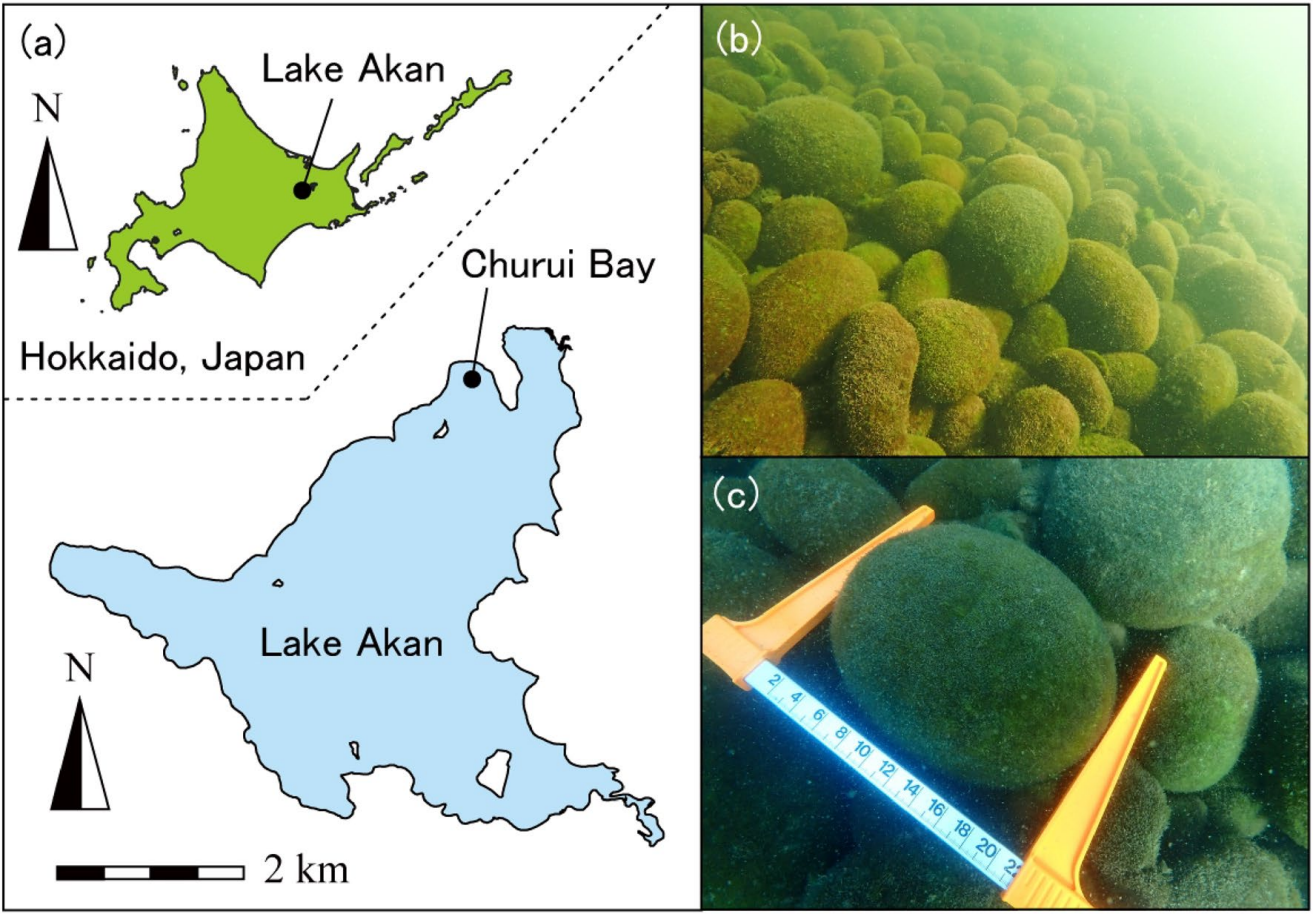
Marimo in Lake Akan. (**a**) Location of Lake Akan and Churui Bay. (**b**) Marimo colony in Churui Bay (26 Aug. 2022). (**c**) Giant Marimo (26 Aug. 2022). Map made by Qgis 3.28.2.

*Aegagropila linnaei* is a filamentous green algal species that is protected in some countries^1–3^. *A. linneaei* often forms beautiful spherical shapes called “lake balls” or, in Japanese, “Marimo”^1^. Marimo can photosynthesize efficiently with a surface thickness of up to 5 cm, forming a central cavity^1,4,5^. Marimo are restricted in their global distribution, and the population has declined worldwide^2,6–11^. For example, eutrophication is a significant reason why Marimo have disappeared in the Netherlands^10^. In 2004, large Marimo colonies existed in Lake Mývatn in Iceland and Lake Akan in Japan, but those in Lake Mývatn had almost disappeared by 2013^3^. Therefore, Lake Akan is the only lake in the world where colonies of giant Marimo (over 20 cm in diameter) remain (Fig. 1). Lake Akan is a caldera lake in eastern Hokkaido Island, Japan; its surface area is 13.28 km^2^ and its maximum depth is 45 m. Giant Marimo grow in depths from 2 to 3 m in the northern portion of Lake Akan. However, sediment deposition caused by lumber transport using rivers extinguished two of the four Marimo colonies in the 1940s^2^. Moreover, the remaining two colonies have been in danger of extinction due to human activities and climate change^5,12–15^. Marimo have a unique nutrient recycling system, which may be the world’s smallest nutrient cycle, supported by the decomposition and mineralization of organic matter^12^. Therefore, the loss of an appropriate physical environment, such as Shiretoko, a World Natural Heritage site, due to climate change may cause irreversible degradation to the nutrient cycles of Marimo and to their ability to sustain themselves^16^.

Marimo are formed by the rotation induced by wind waves, and the physical mechanisms underlying that process have been revealed^4,12,17–19^. Also, to maintain photosynthetic growth by the strong rolling motion of waves, Marimo have been found to shake off the particulate organic matter that accumulates on the surface^12,20,21^. Wave motions also play a great role in polishing the Marimo surface^4,12,18,22,23^. In contrast, Marimo became bushy in a water tank with no movement^24^ and, in the absence of motion, the aggregation of branched uniseriate green algal filaments was revealed to become untangled^18^. Nakayama et al.^12^ summarized the significant factors controlling the formation of giant Marimo:

1. Wind waves due to the land and sea breeze provide sufficient oscillational motion to polish the Marimo surface in a colony during periods with no ice cover^1,19,25^. Moreover, Marimo shake off fine sediments, including particulate organic matter, from their surfaces due to the oscillational motion in order to enable growth by photosynthesis^20,21^.
2. Without motion, the Marimo surface becomes bushy during periods of ice coverage^24^, which generates Marimo’s annual rings.
3. The nutrient recycling system supports the decomposition and mineralization of organic matter from the detached organic matter in the Marimo interior to the exterior surfaces.

Although Marimo balls are well known for their unique spherical shape and growth patterns, the complex interplay between various environmental factors, including hydrological, meteorological, and topographical conditions, makes it difficult to fully understand the mechanisms that govern their growth, including physical aspects. In particular, it remains unknown how Marimo grow to maintain their unique central cavity and thickness due to decomposition by respiration and generation by photosynthesis. A study of internally transcribed spacer ribosomal DNA sequences suggested that *Aegagropila linnaei* or its ancestor is assumed to have dispersed from Central or East Asia^2^. Considering this evidence, immediate conservation action is required to protect the sustainable environment in Lake Akan.

Additionally, the Intergovernmental Panel on Climate Change has stated that stronger wind will occur more in the future, and air temperatures have increased and will continue to increase due to global warming^26–29^. An experiment showed that when Marimo is exposed to water at 35 °C for 24 h, it will fall apart after 80 days^30^. In contrast, The global environmental changes could cause the ice on the lake to be more fragile in the future, indicating that Marimo face a risk of exposure to strong sunlight without a thick ice layer at low temperatures, where photosynthesis is markedly suppressed^31^. Since the spherical shape of Marimo is thought to be maintained by the balance between growth through photosynthesis and decomposition through respiration and other processes, it is very important to evaluate how changes in hydrological and meteorological conditions affect the structure of Marimo.

Therefore, this study aimed to elucidate the decomposition rate in terms of water temperature and the growth rate using annual rings for the existence of giant Marimo. First, we conducted a Marimo decomposition experiment for 289 days to estimate the dry weight density reduction rate under dark conditions. Meanwhile, a magnetic resonance imaging (MRI) analysis revealed the relationship between the cumulative water temperature and the dry weight density reduction rate. Second, an MRI analysis of 10 Marimo sizes from 2 to 20 cm was conducted to find the effects of size on the Marimo growth rate, such as surface thicknesses and annual ring intervals. Finally, we investigated the carbon and nitrogen contents in the surface thickness to show that the growth rate is prominent on the outer side of the surface thickness.

## Results and discussion

### Marimo decomposition under dark condition

**Figure 2:**
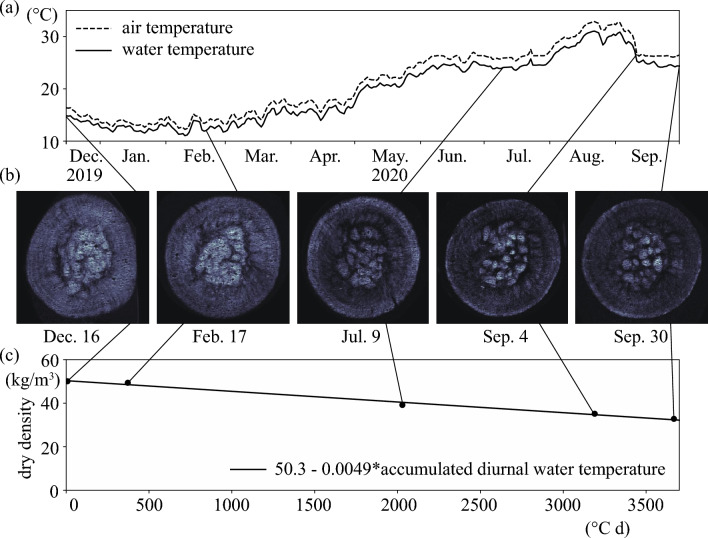
Marimo decomposition experiment. (**a**) Air and water temperature from December 16, 2019, to September 30, 2020. (**b**) MRI of Marimo from December 16, 2019, and February 17, July 19, September 4, and September 30, 2020. (**c**) Accumulated diurnal water temperature from December 16, 2019, to September 30, 2020. The critical water temperature is 7 °C.

The dry weight densities were 50.1, 49.4, 39.2, 35.2, and 32.8 kg/m^3^ on December 16, 2019 and on February 17, July 9, September 4, and September 30, 2020, respectively. Indeed, the MRI values were confirmed to decrease from the beginning to the end of the decomposition experiment, indicating a decrease in the dry weight density (Fig. 2). The dry weight density reduction rate was 0.7 kg/m^3^ for the 2 months from December 16, 2019, to February 17, 2020. However, the reduction was 4.0 kg/m^3^ for the 2 months from July 9 to September 4, 2020, six times the reduction rate in winter. We thus conjectured that the water temperature was a significant factor in controlling the dry weight density. As a result, the dry weight density was negatively proportional to the cumulative water temperature, with a threshold of 7 °C (Fig. 2). The threshold was determined by changing the threshold water temperature from 4 to 15 °C with an interval of 0.1 °C when the highest correlation was obtained. The *r*^2^ was 0.99 with a *p* value of 0.00044 (*N* = 5), which showed a very high correlation and reproducibility. Note that the dry weight density included the detached organic matter in the central cavity, suggesting a necessity for further studies. Additionally, in situ water was not used, and there was no rotational motion in the decomposition experiment, and this lack of motion might have enhanced the decomposition rather than an actual Marimo. Nakayama et al.^12^ revealed that wind waves not only rotate Marimo but also exchange water between the Marimo interior and exterior. The residence time of water inside the Marimo was about 105 h (4.4 d). Since the water in the experimental tank was expected to be hypoxic due to Marimo decomposition, water was exchanged every week to keep the water outside the Marimo aerobic. Since the residence time, 4.4 d, is shorter than the water exchange period, 7 d, of the experiment, in situ water is expected to include more fresh and higher dissolved oxygen than the experiment, suggesting a lower decomposition rate compared to in situ Marimo. Therefore, the dry weight density reduction rate might be underestimated compared to real Marimo.

### Marimo diameter growth rate

**Figure 3:**
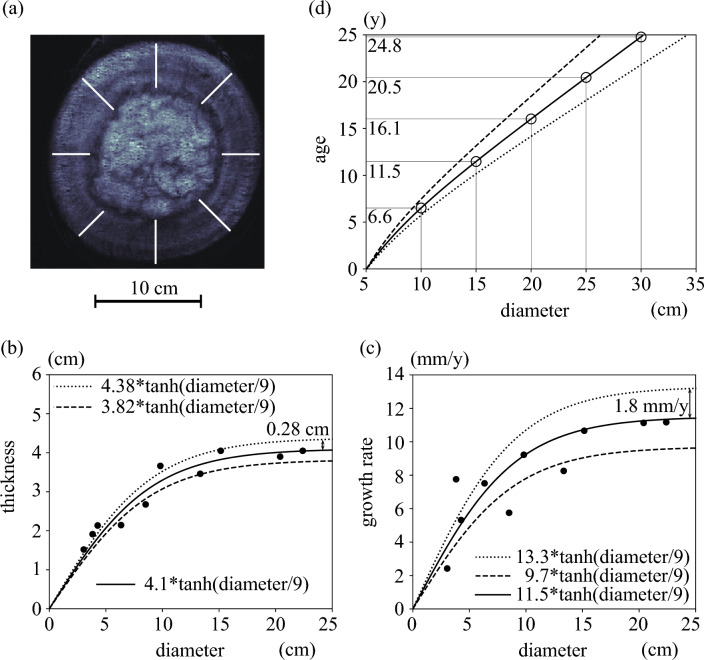
Surface thickness and growth rate. (**a**) MRI image indicating measurement lines. (**b**) Marimo diameter and surface thickness. (**c**) Marimo diameter and diametric growth rate. (**d**) Marimo diameter and estimated age.

Nakayama et al.^12^ revealed that a Marimo has a central cavity with a surface thickness of about 4 to 5 cm, and the specific density ratio decreases as the diameter increases^32^. The lower the specific density ratio, the more the oscillational motion of Marimo is due to the wind wave. Sufficient rotation can provide more effective photosynthesis at the Marimo surface because the underside of the Marimo cannot carry out photosynthesis without rotation of the Marimo. Thus, a larger diameter Marimo is expected to carry out more photosynthesis and have a thicker surface thickness. Therefore, we measured surface thicknesses and annual ring intervals of Marimo with diameters ranging from 3 to 22 cm (Fig. 3). The annual ring interval was converted to the annual Marimo diameter growth rate by following Nakayama et al.^12^, revealing that the annual ring interval corresponds to half the annual growth rate of Marimo diameter. Note that a surface thickness of less than 6 cm was assumed to be half of the Marimo diameter because there was no central cavity. Wind waves are revealed to rotate Marimo the most in June and gradually less from July to October^12^. In Lake Akan, the clarity of the water tends to become the highest in June. And then, it decreases from July to August due to the primary production of the ecosystem, such as the growth of phytoplankton, meaning the photosynthesis activity of Marimo occurs the most in June. Therefore, the coincidence of the Marimo rotation peak and the photosynthesis activity peak may enhance the Marimo growth.

As a Marimo’s surface thickness was expected to reach the limit and maximum value, a hyperbolic tangent function was applied to model the relationship between diameter and surface thickness. Surface thickness was revealed to increase with an increase in diameter (Fig. 3b). This resulted in good agreement, with an *r*^2^ = 0.94 (*p* = 0.0000035). The maximum Marimo surface thickness was estimated to be 4.1 cm with a standard deviation of 0.28 cm. We may have underestimated the maximum Marimo surface thickness because we gave a particular MRI value to distinguish the difference between Marimo and water, implying that the bushy thickness was ignored. Therefore, if we had chopped off a Marimo directly, we may have estimated a greater surface thickness. Similar to Marimo surface thickness, the annual Marimo diametric growth rate increased with an increase in Marimo diameter (Fig. 3c). Again, as the annual Marimo diametric growth rate was expected to reach the limit and maximum value, a hyperbolic tangent function was applied to model the relationship between diameter and that rate. The maximum annual Marimo diametric growth rate was estimated to be 11.5 mm/year, with an *r*^2^ = 0.84 (*p* = 0.0022) and a standard deviation of 1.8 mm/year.

We hypothesized that two main factors contribute to the slower growth rate of Marimo as its diameter becomes smaller. One is the difference in rotational motion due to the size of the Marimo. A numerical simulation showed that smaller Marimo have less resistance to oscillatory currents by wind waves than larger Marimo^19^. Smaller Marimo are polished more easily than larger Marimo, resulting in slower annual Marimo diametric growth and thinner annual rings. The other factor is the placement of Marimo and their growth environment. When the diameter is small, the specific density ratio between the Marimo and water is greater than in the case of a larger Marimo^12^. Therefore, it is highly likely that smaller Marimo are located at the bottom layer of the colony^32^. As a result, water exchange is suppressed, and the water temperature is lower at the bottom layer than at the top of the colony. A laboratory experiment showed that filamentous Marimo grew best at 22 °C water temperatures when incubated from 5 to 22 °C^33^. We measured the water temperature at 0.5 m above Marimo colony and inside Marimo colony with an interval of 10 min from August to November of 2022. The mean temperature was about 0.2 Celsius degrees higher than inside the Marimo colony. Also, the amount of sunlight reaching the bottom layer is limited, which hinders photosynthesis. It is supposed that when the water temperature is low and the amount of sunlight is limited, the growth rate also decreases. Therefore, the larger the Marimo diameter, the moderately higher the water temperature, and the greater the rate of photosynthesis, the faster the annual diametric growth rate and the thicker the Marimo surface. The growth limit of Marimo in Lake Akan is largely controlled by physical factors, e.g., disturbance by typhoons. This is because the maximum thickness of the Marimo is 4–5 cm^12^, so the larger the Marimo, the more fragile it becomes with an increased proportion of the cavity. The largest Marimo recorded in Lake Akan since 2000 is 34 cm in diameter.

The growth of surface thickness on the side of a Marimo was expected to be more active in an actual giant Marimo in nature. Using an estimation formula based on the growth rate of a Marimo using its diameter, it is possible to estimate its diameter and age (Fig. 3d). In Fig. 3d, the initial diameter of the Marimo was assumed to be 5 cm. A smaller Marimo takes 6.6 years to grow from a diameter of 5 to 10 cm because the growth rate is lower than in the case of a larger one. In contrast, it takes 4.6 years for Marimo to grow in diameter from 15 to 20 cm. It was also indicated that it might take between 14.2 and 18.5 years for the diameter to grow from 5 to 20 cm. Note that detailed measurements of water temperature and photon density in the Marimo colony are necessary for future studies.

This study revealed that the maximum annual Marimo diametric growth rate was 11.5 ± 1.8 mm/year. A previous study showed an annual Marimo diametric growth rate range of 9.0 to 12.6 mm/year^12^ using five Marimo with diameters of 6 to 22 cm; the first and second prominent thicknesses of a Marimo annual ring were obtained by the spectral analysis of MRI values of those five Marimo together. Since the amplitudes of MRI values inside a Marimo's surface thickness were lower in smaller than in larger Marimo, it was considered that the interval between Marimo annual rings was obtained mainly in larger Marimo. Therefore, this study's results provide a more detailed and accurate estimation of Marimo annual rings.

### Chemical components and growth of Marimo

**Figure 4:**
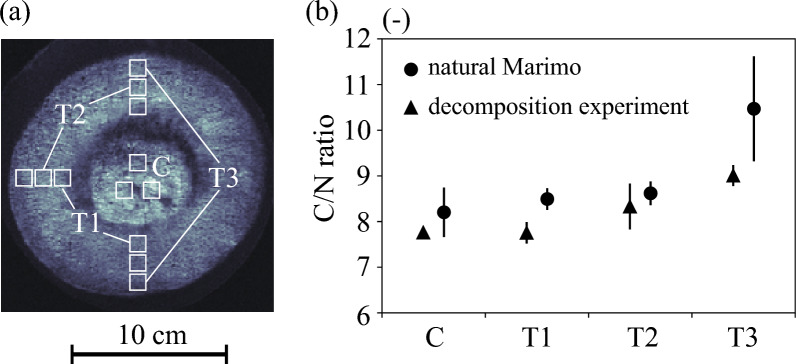
C/N ratio of Marimo. (**a**) Locations of a specimen for measurements. (**b**) C/N ratios at C, T1, T2, and T3 for natural Marimo and the Marimo used in the decomposition experiment.

The carbon contents were almost identical among organic matter in the center (C) and in the interior side, middle, and exterior side of the surface thickness (T1, T2, and T3, respectively) of a natural Marimo, which is not used in the decomposition experiment. However, the nitrogen content was smaller at T3 than at the other areas, resulting in a larger C/N rate at T3 (Fig. 4). The exterior side (T3), that exposed to sunlight, is more easily oxygenated by photosynthesis than the other parts (C, T1 and T2). This leads to the aerobic decomposition of organic matter and mineralization at the exterior side (T3), which are faster for nitrogen in organic matter than for carbon. Similarly, organic matter is less likely to be mineralized in the inner parts, where aerobic decomposition is less active. Therefore, the C/N ratio was lower in the inner parts (C, T1, T2) than in the exterior side (T3), where aerobic conditions are relatively more favorable. In contrast, The C/N ratio of the Marimo used in the decomposition experiment was also higher at T3 than at the other areas, but the difference was almost negligible. This may be due to the exterior side of the surface thickness has acclimatized to the same oxygenated environment as the other areas after a long period of standing in the dark (when photosynthesis is not possible), resulting in a C/N ratio of T3 close to that of C, T1 T2. This result indicates that photosynthesis occurs dominantly at the exterior side of the surface thickness of the Marimo.

Nakayama et al.^12^ conducted field observations to investigate the dissolved oxygen (DO) inside and outside Marimo in 12 samples in August 2015. The mean DO in the Marimo interior was 2.3 mg/L, and the DO outside the Marimo was 8.7 mg/L. Since the mean DO consumption rate was revealed to be 1.9 mg/L/d with a temperature of 27.5 °C and in situ water residence time was 4.4 d in August of 2015, the Marimo interior was hypoxic, suggesting less denitrification inside the Marimo. In contrast, the mean DO consumption rate was 0.67 mg/L/d with a temperature of 11.5 °C in June, and the mean DO in the Marimo interior was 7.8 mg/L, with a DO concentration on the Marimo exterior of 10.5 mg/L. Thus, we confirmed from the field observation in 2015 that the dry weight density reduction rate is strongly associated with water temperature, which supports our Marimo decomposition experiment results.

### Surface thickness estimation using Marimo decomposition and diameter growth rates

**Figure 5:**
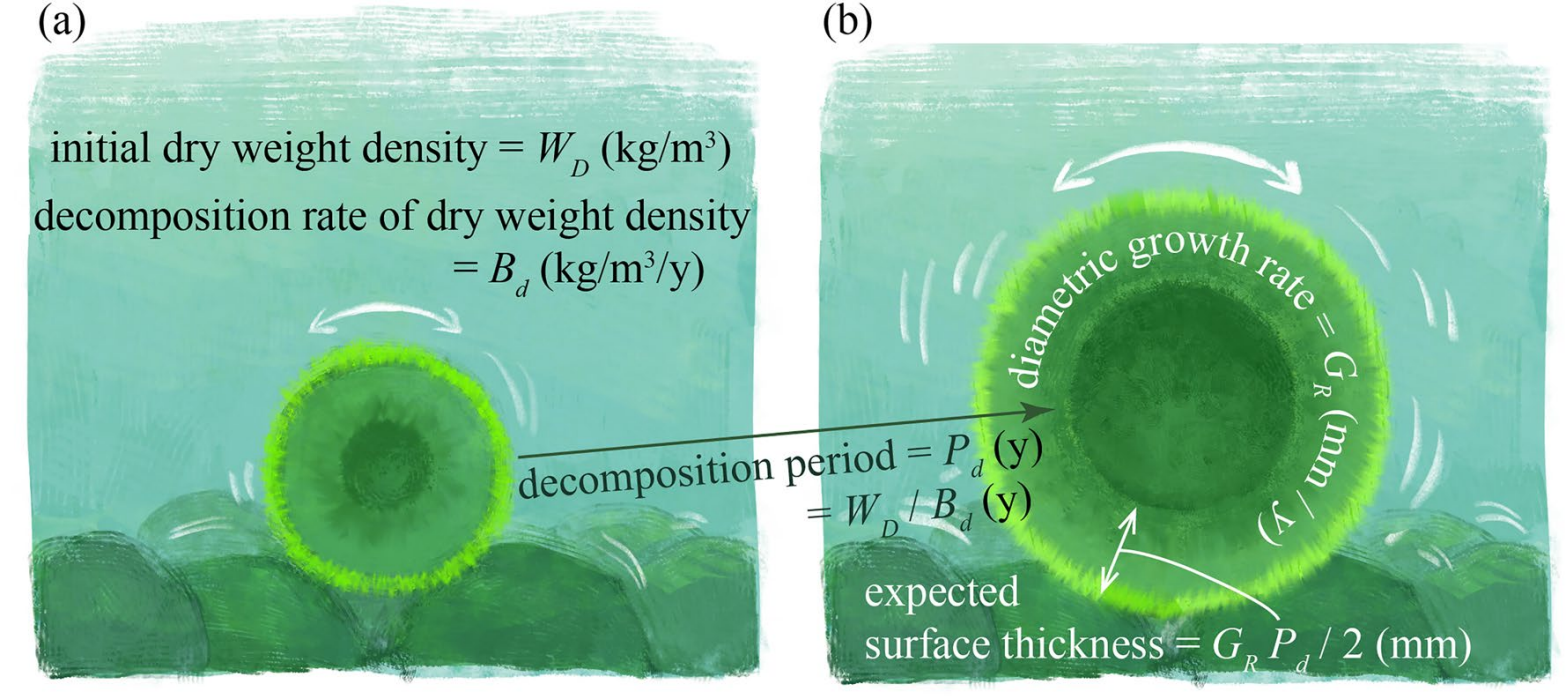
Surface thickness estimation using Marimo decomposition and diameter growth rates. (**a**) Giant Marimo. (**b**) Marimo after decomposition period. Illustration adapted with permission from Reina Nakayama.

The Marimo decomposition experiment showed that the dry weight density of Marimo decreased uniformly at a specific rate as a function of the accumulated water temperature. It also showed that photosynthesis contributed to the growth of the Marimo's exterior surface thickness. Therefore, it may be possible to estimate surface thickness by balancing the Marimo dry weight density loss due to decomposition with the annual Marimo diametric growth rate through photosynthesis. Assuming an initial dry density of ${W}_{D}$ (kg/m^3^) and a decomposition rate of dry weight density decrease of ${B}_{d}$ (kg/m^3^/year), Marimo is expected to decompose in a period of ${P}_{d}={W}_{D}/{B}_{d}$ (y). Since Marimo grows with a diameter growth rate of ${G}_{R}$ (mm/year), the decomposition period should be equal to the growth period, suggesting that the diameter increases by approximately ${G}_{R}{P}_{d}$ (mm). Thus, half the value of the grown diameter ${G}_{R}{P}_{d}/2$ (mm) is the surface thickness (Fig. 5). Noted that the region of the Marimo that decomposes in ${P}_{d}$ (y) corresponds to the central cavity.

To estimate the surface thickness, it is necessary to calculate the accumulated water temperature to obtain the decomposition rate of Marimo dry weight density. The Marimo samples used for MRI analysis were collected between 2017 and 2019. To form a surface thickness of 4.1 ± 0.28 cm, the maximum diameter growth rate is assumed to be 11.5 ± 1.8 mm/year, requiring a minimum period of 7.0 (= 2*4.1/1.15) years. The accumulated water temperature from 2010 (= 2017-7) or 2012 (= 2019-7) to 2019 is necessary to estimate the surface thickness of the samples between 2017 and 2019 for comparison with the maximum surface thickness of 4.1 ± 0.28 cm. However, water temperature measurements around the Marimo colony were not taken during the target period. Since water temperature changes were measured in the Marimo colony only from 1988 to 1990 and from 2021 to 2022, the accumulated water temperature from 2012 to 2019 was similarly assumed using the data from 2021 to 2022. Then, the accumulated water temperature in the Marimo colony in 2021 was 1780 °C-days Celsius (°C d). As 2021 was a hot year, with an average temperature 0.9 °C higher than the mean temperature from 2012 to 2019, the accumulated water temperature from 2012 to 2019 is estimated to be 1609 °C d (= 1780–0.9*190). Thus, the decomposition rate of Marimo dry weight density from 2012 to 2019 is estimated to be 7.88 kg/m^3^/year. Given that the initial density of Marimo is 50.3 kg/m^3^, the decomposition period is calculated to be 6.4 (= 50.3/7.88) years. As a result, the maximum surface thickness estimated using an annual Marimo diametric growth rate of 11.5 ± 1.8 mm/year is 3.7 (= 1.15*6.4/2) cm, consistent with the maximum value of 4.1 ± 0.28 cm obtained by MRI analysis. However, the initial Marimo dry weight density obtained from the decomposition experiment may be underestimated, as mentioned above. In other words, the thickness of 3.7 cm may be an underestimation, suggesting that the estimated surface thickness may be consistent with the actual phenomenon.

Decades ago, the cumulative water temperature in the Marimo colony in 1988 was 1250 °Cd. This means that the annual decrease in the dry density of Marimo was estimated to be 6.1 (= 1250*0.0049) kg/m^3^/year. Marimo was found to decompose in approximately 8.2 (= 50.3/6.1) years. Using an annual diametric growth rate of 11.5 ± 1.8 mm/year, the maximum surface thickness is estimated to be 4.7 (= 1.15*8.2/2) cm. In 1988, the cumulative water temperature was lower than in 2021, and conditions were in place to form a surface thickness of about 1 cm greater than in the current situation.

To maintain a Marimo with a surface thickness of 4.1 ± 0.28 cm, it is estimated that it would take at least approximately 7 years (= 2*4.1/1.15) by utilizing the maximum annual Marimo diametric growth rate of 11.5 ± 1.8 mm/year over a year. Therefore, the decomposition rate of the dry weight density of Marimo must be below 7.2 kg/m^3^/year (= 50.3/7.0). The decomposition rate of 7.2 kg/m^3^/year corresponds to a cumulative water temperature of 1469 °C-days (= 7.2/0.0049). Therefore, it is considered that the optimal environment for giant Marimo to grow requires a cumulative water temperature of approximately 1470 °C-days or less. Spherical Marimo greater than 15 cm in diameter have been confirmed only in relatively cold regions, such as Lake Zeller in Austria^34^, Lake Hederviken in Sweden^4^, and Lake Saadjärve in Estonia^35^. In warmer regions, water temperatures from April to December are above 7 °C. They can reach up to approximately 28 °C at peak times, so the cumulative water temperature over a year may exceed 3000 °C-days. Therefore, it is estimated that the decomposition rate of Marimo dry weight density over a year must be at least approximately 15.0 (= 3000*0.0049) kg/m^3^/year. As a result, to maintain a relatively thin surface thickness of 3.0 cm, a diametric growth rate of 18.0 mm/year (= 2*30/(50.3/15.0)) is expected to be needed. In a cultivation experiment using an artificial medium for 5 months, filamentous Marimo showed annual growth of 4.0 mm at a water temperature of 10 °C and 6.0 mm at a water temperature of 14 °C^36^. Based on the average water temperature of approximately 11 °C for 8 months in 1988, excluding when Lake Akan is frozen from January to April, filamentous Marimo are expected to grow by 6.4 to 9.4 mm in 8 months. A Marimo has fewer nutrients in the natural environment than in artificial medium and, because its surface is polished by rotation, it is impossible to secure an artificial medium and thus impossible to achieve an annual growth rate of 18.0 mm/year in diameter. Therefore, Marimo is assumed to survive only in relatively cold regions.

Meteorological observation data from the Automated Meteorological Data Acquisition System (AMeDAS) for Lake Akan from 1980 to 2020 show that the annual average temperature increased by about 2.0 °C in that period. The rising water temperature is likely due to the effects of global warming, and if this trend continues, it may become difficult to maintain a healthy surface thickness. Rivers with water colder than lake water, flowing into the Churui Bay and Marimo growth areas of Lake Akan^37^, could be utilized to minimize the effects of global warming on Marimo by taking advantage of the cooler water.

## Conclusion

A decomposition experiment on Marimo revealed that the dry weight density of Marimo has a high correlation with cumulative water temperature with a threshold of 7 °C. Using 10 Marimo ranging in diameter from 3 to 22 cm, we found that a hyperbolic tangent function can estimate the thickness and annual growth rate, revealing the relationship between Marimo's diameter and age. On the other hand, the C/N ratio was found to be high in the exterior side of the surface thickness, indicating that this layer is the main growth area for photosynthesis. The exterior side is exposed sunlight and is more easily oxygenated by photosynthesis than the other parts. These results suggest that the central cavity and the surface thickness represent the change in the growth environment such as water temperature and light intensity. Between the 1980s and the present, the cumulative water temperature has increased from about 1250 to about 1600 °C-days. There is a possibility that the maximum surface thickness has decreased by about 1 cm. If global warming continues and the water temperature rises, giant Marimo could become extinct. Therefore, it is necessary to propose measures to protect Marimo from global warming, such as utilizing the cooler river water that flows into the Marimo colony.

## Materials and methods

### Marimo decomposition under dark condition

We conducted a Marimo decomposition experiment for 289 days from December 16, 2019, to September 30, 2020, using an acrylic tank with plane sizes of 40 cm and 40 cm and a height of 50 cm to estimate the dry weight density reduction rate under a condition of darkness. The water depth was kept at 40 cm, and the dark condition were used at ambient temperature. The diameter of the Marimo was 15 cm. Since all of the water in the acrylic tank was expected to become anoxic due to Marimo decomposition, water in the tank was exchanged every week to keep the water outside the Marimo aerobic. The exchange water was kept for 2 days at ambient temperature before it replaced the water in the tank in order to avoid a temperature difference. Water temperature was measured inside the acrylic tank at an interval of 10 min.

MRI (Intera Achieva 1.5 T Nova Dual; Philips) analysis was performed to measure 3D dry weight density five times: on December 16, 2019, and on February 17July 9, September 4, and September 30, 2020. We placed the Marimo into a polyethylene bag filled with water for MRI analysis of the void ratio. Then, a proton density-weighted image was obtained using a head coil, which showed that the higher the density of green algal filaments, the larger the MRI value. Three Marimo samples were collected to convert MRI values to a Marimo dry weight density. The three samples were dried by using a constant-temperature drying oven (EYELA, NDO-400). The sample volumes were 5009 mm^3^, 5954 mm^3^, and 8346 mm^3^ from inner to outer surface thickness. Vernier calipers were used to measure the sample volumes with a confidence interval of 1 mm. The sample weights were 0.1732, 0.1704, and 0.3214 g, according to the volumes. Therefore, the dry weight densities were obtained as 34.59, 28.62, and 38.50 kg/m^3^. The MRI values were converted to dry weight densities using the calibration curve obtained by the three samples. Note that there was no MRI image between February 17 July 9, 2020, because entrance to Kobe University Hospital was restricted at that time due to COVID-19.

### Marimo diameter growth rate

The 3D dry weight density distribution was calculated according to MRI values (proton density-weighted images) that were obtained using the calibration curve between an MRI value and dry weight density. The calibration curve between an MRI value and dry weight density was made using nine samples shown in Fig. 4 (three samples each for T1, T2 and T2). A proton density-weighted image shows that the higher the density of green algal filaments, the larger the MRI value. Undoubtedly, black corresponds to zero dry-weight density. The MRI slice images were obtained every 1.5 mm interval, meaning there are more than 100 slice images for a 15 cm diameter giant Marimo, enabling us to estimate the 3D dry weight density by integrating the MRI values. In each MRI slice image, the grid resolution was 0.5 mm with 512 and 512 grid sizes, covering 25.6 cm for horizontal and vertical sides.

The annual Marimo diameter growth rate was estimated from the sparse and dense thickness of the MRI values along the radial direction^12^. Since the growth rate of Marimo varies by the growth stage and environmental conditions, each sparse and dense thickness has variable values. Therefore, the spectrum using the MRI values along the radial direction was obtained to investigate the predominant thickness of the annual rings^12^. The first peak of the spectrum was used to estimate the annual Marimo diameter growth rate.

### Chemical components and growth of Marimo

According to the 3D dry weight density distribution of Marimo, photosynthesis was expected to occur mainly at the exterior side of the surface thickness. Therefore, we used an organic elemental analyzer (Perkin Elmer, 2400II) to investigate the relationship between carbon and nitrogen and an annual Marimo diametric growth rate. We used two Marimo in this analysis. One is a Marimo shortly after collection from the habitat, the other is a Marimo after the decomposition experiment for 289 days. Three samples were obtained from detached organic matter in the center (C) and the interior side, middle, and exterior side of the surface thickness (T1, T2, and T3, respectively) of the two Marimo, respectively (Fig. 4). We cut off all samples with each volume of about 6 cm3 and ground the dried specimen in a mortar by using alumina pestle. The specimen was dried using the constant temperature dryer (EYELA, NDO-400).

## Data Availability

The data that support the findings of this study are available from the corresponding author upon reasonable request.
